# Apelin-13 Increases Functional Connexin-43 through Autophagy Inhibition via AKT/mTOR Pathway in the Non-Myocytic Cell Population of the Heart

**DOI:** 10.3390/ijms232113073

**Published:** 2022-10-28

**Authors:** Emanuela Vitale, Rachele Rosso, Marco Lo Iacono, Caterina Cristallini, Claudia Giachino, Raffaella Rastaldo

**Affiliations:** 1 Department of Clinical and Biological Sciences, University of Turin, 10043 Orbassano, Italy; 2 Institute for Chemical and Physical Processes, IPCF ss Pisa, CNR, 56126 Pisa, Italy

**Keywords:** connexin-43, apelin-13, non-myocytic cells, autophagy, AKT/mTOR pathway, gap junction, LC3, p62, H9C2 cells

## Abstract

Studies have shown a link between the downregulation of connexin 43 (Cx43), the predominant isoform in cardiac gap junctions, and high susceptibility to cardiac arrhythmias and cardiomyocyte death. Non-myocytic cells (NMCs), the most abundant component of the heart, exert multiple cardiac functions and represent an important therapeutic target for diseased cardiac tissue. A few studies have investigated the effect of Apelin-13, an endogenous peptide with a key role in various cardiovascular functions, on Cx43 expression in cardiomyocytes. However, it remained unknown whether Apelin-13 influences Cx43 expression in NMCs. Here, we found that in NMCs, Cx43 protein expression increased after Apelin-13 treatment (100 nM for 48 h). Furthermore, dye transfer assays proved that Apelin-13-treated NMCs had a greater ability to communicate with surrounding cardiomyocytes, and this effect was abrogated by carbenoxolone, a gap junction inhibitor. Interestingly, we showed that Apelin-13 increased Cx43 through autophagy inhibition, as proved by the upregulation of p62 and LC3I, acting as 3-MA, a well-known autophagy inhibitor. In addition, Apelin-13-induced AKT and mTOR phosphorylation was abolished by LY294002 and rapamycin inhibitors resulting in Cx43 increased suppression. These results open the possibility of targeting gap junctions in NMCs with Apelin-13 as an exciting therapeutic approach with great potential.

## 1. Introduction

Cardiovascular diseases (CVDs) are a group of disorders that nowadays constitute the leading cause of death worldwide [1,2]. Some of the most relevant cardiac diseases, such as myocardial infarction, heart failure, and atrial fibrillation, are characterised by altered connexin expression and gap junction remodelling that take part in increasing the susceptibility to cardiac arrhythmias and cardiomyocyte death [3,4,5].

Connexins are transmembrane glycoproteins trafficking on the cell membrane; six protein subunits make up a hemi-channel called connexon, which, in turn, docks with another one on the plasma membrane of the adjacent cell, thus forming the gap junction channels. These cell–cell connections allow the passage of ions from one cell to the next, enabling the transmission of the electrical impulse that is essential for cardiac function [6,7]. Connexin-43 (Cx43) is the primary isoform expressed in mammalian cardiac tissue [4,8]. It is well-known that Cx43 dysregulation can compromise the electrical properties and homeostasis of cardiac tissue as it occurs in pathological conditions (such as hypertrophic, ischemic, and diabetic hearts) [3,7].

Within the heart, cardiomyocytes account for the 30% of cellular population, but the most abundant cardiac component is made up of non-myocytic cells (NMCs) [9,10,11]. Resident NMCs include fibroblasts, endothelial cells, immune cells, and smooth muscle cells. In addition, they contain resident stem cells partially committed to the cardiac lineage [12]. Importantly, NMCs are positive for Cx43 [11,12]. NMCs are important players in cardiac homeostasis and disease, exhibiting multiple central functions in the heart such as vascular supply and production of the extracellular matrix and contributing to cardiomyocyte survival. Indeed, NMCs provide structural, biochemical, mechanical, and electrophysiological properties that are necessary for the maintenance of heart structure and function [11,13,14] at least in part by entering in direct contact with cardiomyocytes through gap junctions [11].

Autophagy is an evolutionary conserved mechanism through which cytoplasmatic elements are internalised by autophagosomes and degraded or recycled contributing to the cellular homeostasis [15]. In the heart, autophagy is known to be one of the principal regulators of cardiac homeostasis and functionality through the elimination of damaged cellular components, providing substrates for ATP renewal during ischemia and through the reduction of progressive dysfunction and remodelling with the inhibition of mitochondrial dysfunction, misfolded proteins, and oxidative stress [16]. However, while the basal levels of autophagy are physiological and important in the regulation of cardiac homeostasis, when autophagy is excessively activated, it can induce apoptosis, thereby aggravating cardiac damage, hypertrophy, myocardial infarction, diabetic cardiomyopathy, and heart failure [16,17,18]. Recently, autophagy has emerged as the main regulation and degradation process of Cx43 [19]. A few studies have shown that among the effects derived from the increase in autophagic flux, there is the downregulation of Cx43 in cardiomyocytes and H9C2 cells [20,21,22].

Apelin is an endogenous peptide produced by various tissues, and it acts on its G protein-coupled receptor called APJ. Among various isoforms, Apelin-13 is the most abundant and potent in the heart [23]. Apelin-13 displays several important effects on the cardiovascular system: it is involved in cardiac development; mechanical function increasing cardiac contractility in both healthy and failing hearts; protective activity preventing ischemia–reperfusion injury, cardiac fibrosis, and remodelling; as well as the regulation of blood pressure inducing vasodilation [23,24]. Previous evidence suggested that Apelin-13 could be involved in the regulation of Cx43 in neonatal rat cardiomyocytes and HL-1 cells, a cardiac myocyte cell line [25,26]. However, until now, the role of Apelin-13 on possible Cx43 regulation in NMCs has remained unexplored.

In this study, we investigated for the first time whether the treatment of NMCs with Apelin-13 was able to increase Cx43 protein levels and intercellular communication, whether the possible effect exerted by Apelin-13 took place through autophagy modulation, and what signalling pathway was involved.

## 2. Results

### 2.1. NMCs Treated with Apelin-13 Increase Connexin-43 Protein Levels and Functional Gap Junction Formation

**Figure 1:**
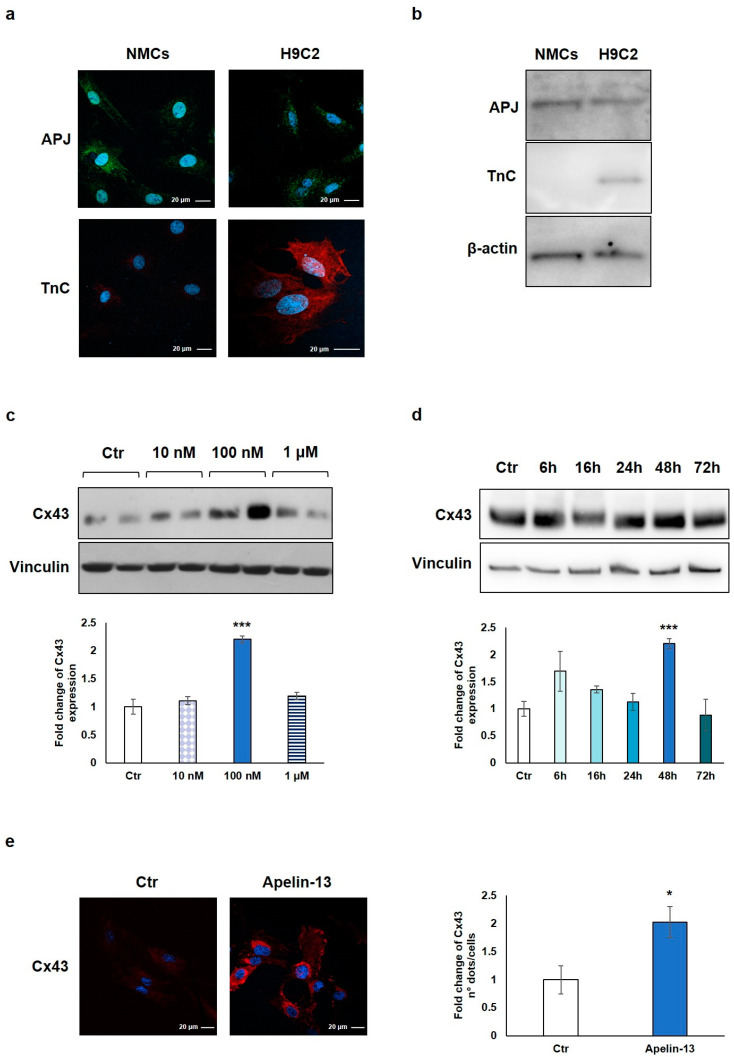
Apelin-13 treatment increases Cx-43 protein level in NMCs. (**a**) Immunofluorescence evaluation of APJ receptor and TnC expression in NMCs and H9C2 cells. Apelin-13 receptor was stained with anti-APJ antibody (green), and TnC was stained with anti-TnC antibody (red). Cell nuclei were visualised using Hoechst (blue). Images were acquired by confocal laser microscopy at 63×, scale bar 20 μm. (**b**) APJ receptor and TnC expression was also assessed by Western blotting in both NMCs and H9C2 cells; β-actin served as an internal. (**c**) Western blot analysis of Cx43 protein expression in NMCs after cell treatment with different doses of Apelin-13 (10 nM, 100 nM, and 1 μM) for 48 h. Quantitative evaluation of band intensity was expressed as fold change of Apelin-13-treated NMCs vs. untreated cells (Ctr). Vinculin served as internal control (mean ± SEM; *** *p* ≤ 0.001; *n* = 3). (**d**) Western blot analysis of Cx43 protein level after NMC treatment with 100 nM Apelin-13 for different timing (6 h, 16 h, 24 h, 48 h, and 72 h). Quantitative evaluation of band intensity was expressed as fold change of Apelin-13-treated NMCs vs. Ctr cells. Vinculin served as internal control (mean ± SEM; *** *p* ≤ 0.001; *n* = 3). (**e**) Immunofluorescence analysis of Cx43 protein expression in Apelin-13-treated NMCs (100 nM, 48 h). Cx43 was stained with anti-Cx43 antibody (red), and cell nuclei were visualised using Hoechst staining (blue). Quantitative evaluation of fluorescent dots/cell intensity was expressed as fold change of Apelin-13-treated NMCs vs. Ctr cells (mean ± SEM; * *p* ≤ 0.05; *n* = 3). Images were acquired by confocal laser microscopy at 63×, scale bar 20 μm.

We first verified the expression of the APJ receptor on freshly isolated rat NMCs and, as control, on H9C2 cells, a cardiomyoblast cell line used as an in vitro model for its similarity with primary cardiomyocytes. Immunofluorescence and Western blot analyses showed the expression of APJ in both NMCs and H9C2 cells (Figure 1a,b). The expression of troponin C (TnC) was verified as cell lineage identity control and, as expected, only H9C2 cells turned out to be positive, demonstrating that the isolated NMCs do not contain any residual cardiomyocyte (Figure 1a,b).

NMCs were then treated with Apelin-13 at the indicated concentrations (10 nM, 100 nM, and 1 µM) for 48 h in order to investigate the possible Cx43 modulation. The results of the Western blot analysis demonstrated that Apelin-13 treatment significantly (*p* = 0.00049) increased Cx43 protein levels at 100 nM concentration (Figure 1c), and the effect disappeared at a higher dose (1 µM). To evaluate if 48 h was the best incubation time for our experiments, we performed a time course analysis with 100 nM Apelin-13 observing protein levels after 6, 16, 24, 48, and 72 h of treatment. Data showed that after treatment with Apelin-13, the Cx43 protein level resulted significantly (*p* = 0.00049) enhanced at 48 h (Figure 1d), whereas after 72 h, it returned to a value similar to untreated cells. Based on these results, the treatment of NMCs with 100 nM Apelin-13 for 48 h represented the optimal experimental condition, and it was reproduced in all the subsequent experiments.

Immunofluorescence and confocal microscopy analysis allowed to confirm the Apelin-13-induced upregulation of Cx43 in NMCs. Qualitative observation of images suggested that Apelin-13 treatment increased Cx43 expression; quantitative evaluation confirmed the significant (*p* = 0.0316) increase in Cx43 expression in Apelin-13-treated NMCs compared with control cells (Figure 1e).

**Figure 2:**
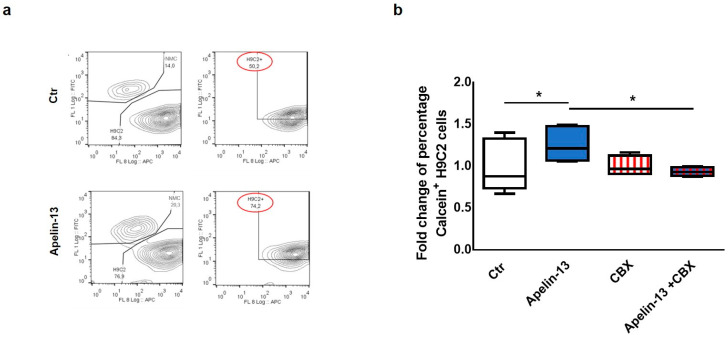
Apelin-13 treatment augments intercellular communication through gap junctions. (**a**) Representative dye transfer experiment in flow cytometry. Upper panel: H9C2 cells labelled with Did dye (APC) were cultured for 24 h with control NMCs labelled with Calcein-AM (FITC). Lower panel: H9C2 cells were cultured for 24 h with NMCs treated with Apelin-13 and labelled with Calcein-AM. After coculture, H9C2 cells double-positive (APC+ FITC+) were analysed through flow cytometry; green (FITC+) fluorescence detected in H9C2 cells was due to Calcein-AM they received through intercellular communication with NMCs. (**b**) Dye transfer analysis of H9C2 cells cocultured for 24 h with NMCs treated or untreated with Apelin-13 and in the presence or absence of gap junction inhibitor carbenoxolone (CBX). Before treatment, NMCs were stained with Calcein-AM (gap junction permeant dye), whereas H9C2 cells were stained with Did dye to discriminate the two types of cells. Boxplot shows distribution of fold change of mean fluorescence relative to Calcein + cells. At least 80,000 cells were analysed for each experiment (* *p* ≤ 0.05; *n* = 3).

Starting from our experimental evidence that Apelin-13 was able to increase the expression of Cx43 in NMCs, we investigated the possibility that NMCs treated with Apelin-13 might have an improved capability to communicate with cardiac cells via gap junctions. Therefore, in order to test the role of Apelin-13 on gap junction functionality, a dye transfer assay was performed. In particular, we evaluated the NMCs’ ability to transfer the gap junction permeant dye Calcein-AM (FITC) to H9C2 cells labelled with Did dye (APC). Control or Apelin-13-treated NMCs were stained with Calcein-AM and subsequently seeded in coculture with H9C2 cells for 24 h. Cytofluorimetric analysis was then performed in order to identify the FITC+APC+ double-positive H9C2 cells. The FITC fluorescence detected in H9C2 cells could be exclusively due to the Calcein-AM they received from NMCs through gap junction-mediated intercellular communication. The results obtained showed that the percentage of Calcein+ H9C2 cocultured with Apelin-13-treated NMCs was significantly higher, with an increase of about 50% of green cells with respect to H9C2 cocultured with untreated NMCs (74.2% vs. 50.2%, respectively, *p* = 0.0295, Figure 2a,b). These data suggested that Apelin-13 improved the ability of NMCs to transfer the dye to H9C2 cells and, therefore, to exchange small molecules and ions with the surrounding cells. In order to assess whether the increase in dye transfer after Apelin-13 treatment occurred via the gap junction only, Apelin-13 treatment was performed also in the presence of 100 nM carbenoxolone (CBX), a specific gap junction inhibitor. As expected, the results of the cytofluorimetric analysis showed that CBX was able to significantly (*p* = 0.0185) suppress the positive effect of Apelin-13 in increasing dye transfer from NMCs to H9C2 cells (Figure 2b). These data strongly suggest that the more efficient cell-to-cell communication is ascribable to the formation of gap junctions due to higher Cx43 protein levels.

### 2.2. Apelin-13 Increases Cx43 Protein Levels through Autophagy Inhibition

**Figure 3:**
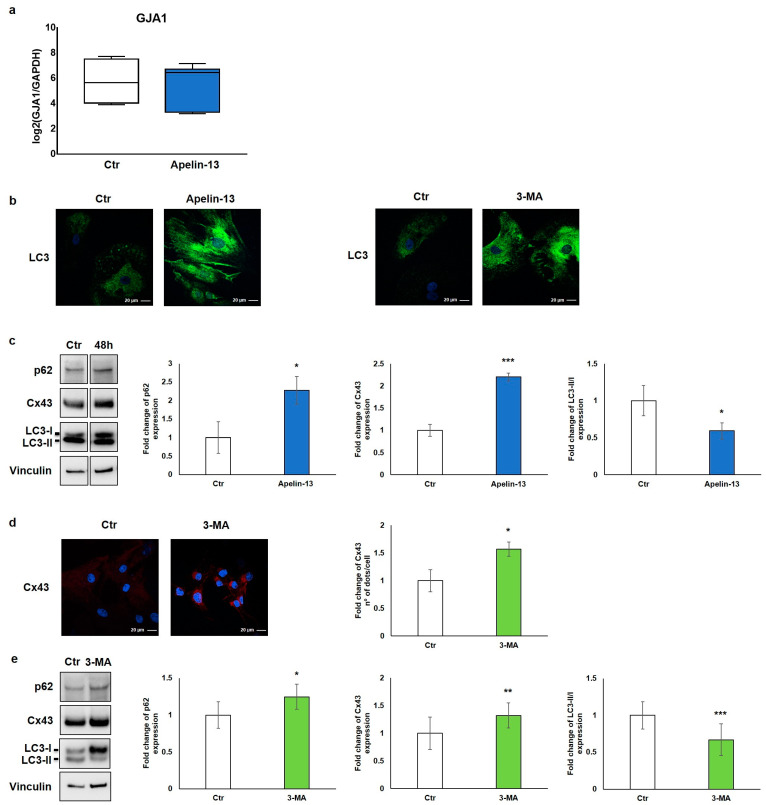
Apelin-13 increases Cx43 protein levels through autophagy inhibition. (**a**) Quantitative real-time PCR of GJA1 gene in NMCs untreated or treated with Apelin-13 (*n* = 3). (**b**) Qualitative immunofluorescence evaluation of LC3 expression in Apelin-13- and 3-MA-treated NMCs vs. Ctr cells. LC3 was stained using anti-LC3 antibody (green), and cell nuclei were visualised using Hoechst (blue). Images were acquired by confocal laser microscopy at 63×, scale bar 20 μm. (**c**) Western blot analysis of p62, Cx43, and LC3 protein expression in NMCs after Apelin-13 treatment (100 nM, 48 h). Quantitative evaluation of band intensity was expressed as fold change of Apelin-13-treated NMCs vs. Ctr cells. Vinculin served as internal control (mean ± SEM; * *p* ≤ 0.05; *** *p* ≤ 0.001; *n* = 4). (**d**) Immunofluorescence analysis of Cx43 protein expression in 3-MA-treated NMCs (5 mM, 16 h). Cx43 was stained with anti-Cx43 antibody (red), and cell nuclei were visualised using Hoechst (blue). Images were acquired by confocal laser microscopy at 63×, scale bar 20 μm. Quantitative evaluation of dots/cell intensity was expressed as fold change of 3-MA-treated NMCs vs. Ctr cells (mean ± SEM; * *p* ≤ 0.05; *n* = 3). (**e**) Western blot analysis of p62, Cx43, and LC3 protein expression in NMCs after 3-MA treatment. Quantitative evaluation of band intensity was expressed as fold change of 3-MA-treated NMCs vs. Ctr cells. Vinculin served as internal control (mean ± SEM; * *p* ≤ 0.05; ** *p* ≤ 0.01; *** *p* ≤ 0.001; *n* = 3).

In order to elucidate the biological mechanism leading to the increased Cx43 protein expression in NMCs treated with Apelin-13, the expression of gap junction alpha-1 (*Gja1*), the gene encoding for connexin-43, was transcriptionally evaluated. Collected data showed that the relative expression of the *Gja1* gene in NMCs treated with 100 nM Apelin-13 for 48 h did not differ compared with control cells (Figure 3a), suggesting that Apelin-13 does not affect *Gja1* gene expression. Accordingly, to establish through which mechanism Apelin-13 induces the increase in the Cx43 protein level, with consequent improvement in the gap junction function, we indagated its protein turnover.

Since one of the main processes of regulation and degradation of Cx43 is autophagy [19], we investigated the involvement of this molecular pathway. We thus studied control and Apelin-13-treated NMCs for the expression of the autophagic marker microtubule-associated protein 1 light-chain 3 (LC3). LC3 is essential for autophagosome biogenesis and maturation [27]. There are two forms of LC3, called LC3-I and LC3-II; the former is the cytosolic form, whereas the latter is the membrane-bound form conjugated to phosphatidylethanolamine, which is associated to the autophagosome [27,28]. The results of the qualitative immunofluorescence analysis revealed that NMCs treated with Apelin-13 showed a high LC3 cytosolic protein level compared with that of the control cells (Figure 3b, left panel). A comparable result was also observed when NMCs were treated with 3-methyladenine (3-MA), a well-known autophagy inhibitor (Figure 3b, right panel).

Starting from the evidence that Apelin-13 was able to increase the LC3 amount in a way comparable with an autophagy inhibitor, we hypothesised that Apelin-13 could similarly increase Cx43 levels through the inhibition of the autophagic flux. To investigate the role of Apelin-13 in autophagy, we performed a Western blot analysis in which we evaluated Cx43, the LC3-II/LC3-I ratio, and p62 protein, also called Sequestosome 1. The autophagy receptor p62 serves to link ubiquitinated proteins to the autophagic machinery, and it is itself degraded by autophagy. Since p62 accumulates when autophagy is inhibited, while lowered levels can be seen when autophagy is induced, p62 as well as LC3 may be used as valuable markers to study the autophagic flux.

The results of the Western blot analysis showed that Apelin-13 not only increased the expression of Cx43 (*p* = 0.00049) but also augmented p62 (*p* = 0.050) and LC3-I levels as demonstrated by the significant reduction of the LC3-II/LC3-I ratio (*p* = 0.0292) (Figure 3c). These data suggest that Apelin-13 could exert its action impairing the autophagy pathway. To reinforce our hypothesis, Cx43 protein levels were evaluated after NMC treatment with a 3-MA autophagy inhibitor. The results of the immunofluorescence analysis showed that Cx43 was significantly (*p* = 0.0302) increased when NMCs were treated with 3-MA compared with the control (Figure 3d). In addition, the results of the Western blot analysis revealed that 3-MA, beyond modulating the p62 and LC3 autophagy markers, as expected, significantly increased Cx43 in NMCs (*p* = 0.0127, *p* = 0.00015, and *p* = 0.009, respectively, Figure 3e). Taken together, these results hint that Apelin-13, as wellas 3-MA, was capable of altering the autophagic flow, thus leading to increased levels of Cx43 protein in NMCs.

### 2.3. Akt/mTOR Pathway Is Responsible for Apelin-13-Mediated Autophagy Inhibition and Cx43 Increase

**Figure 4:**
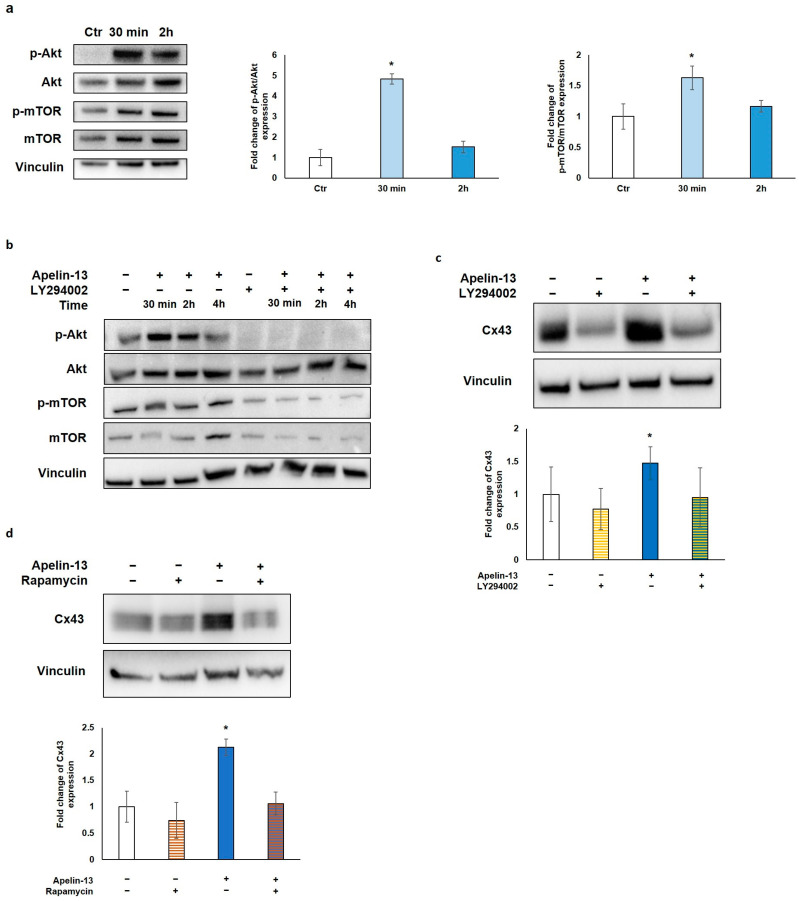
Apelin-13 increases Cx43 via Akt/mTOR-dependent autophagic pathway. (**a**) Western blot analysis of pathway activated in NMCs by 100 nM Apelin-13 treatment for 30 min and 2 h. Central graph shows quantification of p-Akt/Akt protein expression in Apelin-13-treated NMCs. Vinculin served as internal control (mean ± SEM; * *p* ≤ 0.05; *n* = 6). Graph on the right shows quantification of p-mTOR/mTOR protein expression in Apelin-13-treated NMCs (mean ± SEM; * *p* ≤ 0.05; *n* = 7). Quantitative evaluation of band intensity was expressed as fold change of Apelin-13-treated NMCs vs. Ctr cells. (**b**) Western blot evaluation of effect of 100 nM Apelin-13 for 30 min, 2 h, and 4 h alone or in combination with 10 μM LY294002 inhibitor. Vinculin served as internal control. (**c**) Western blot analysis of Cx43 protein expression in Apelin-13-treated (100 nM, 48 h) or untreated NMCs with or without LY294002 inhibitor. Quantitative evaluation of band intensity was expressed as fold change of Cx43 protein expression in treated vs. untreated cells. Vinculin served as internal control (mean ± SEM; * *p* ≤ 0.05; *n* = 4). (**d**) Western blot analysis of Cx43 protein expression in NMCs either in the presence or absence of p-mTOR inhibitor rapamycin. Cells were treated with inhibitors alone or in combination with 100 nM Apelin-13 for 48 h. Quantitative evaluation of band intensity was expressed as fold change of Cx43 protein expression in treated vs. control cells. Vinculin served as internal control (mean ± SEM; * *p* ≤ 0.05; *n* = 3).

To investigate the molecular mechanism through which Apelin-13 affects autophagy, we explored the role of the protein kinase B/mammalian target of rapamycin (Akt/mTOR) pathway, which is strongly linked to autophagy modulation. In fact, Akt, a downstream effector molecule of phosphatidylinositol 3-kinase (PI3K), negatively regulates autophagy by targeting mTOR, which, in turn, inhibits multiple autophagy-promoting proteins. Western blot analysis of p-Akt/Akt and p-mTOR/mTOR protein levels was performed after NMC treatment with Apelin-13 for 30 min and 2 h. Our results showed that after 30 min of treatment with Apelin-13, both p-Akt/Akt and p-mTOR/mTOR resulted to be significantly increased compared with the control (*p* = 0.0345 and *p* = 0.0327, respectively), whereas both phosphorylation levels returned back close to the control value after 2 h (Figure 4a). These data suggest that the Apelin-13-induced increases in Cx43 protein expression can be due to Akt/mTOR pathway activation. To further investigate the involvement of Akt/mTOR in this process, NMCs were treated with Apelin-13 in combination or not with LY294002, a well-known highly selective inhibitor of PI3K-dependent Akt phosphorylation. Once it was verified that LY294002 was able to inhibit Akt phosphorylation and, in turn, phosphorylation of mTOR in both control cells and in Apelin-13-treated NMCs (Figure 4b), we analysed Cx43 protein levels in the presence of LY294002. Western blot revealed that when this inhibitor was used for 48 h together with Apelin-13, it was able to reverse the Apelin-13-induced Cx43 increase, keeping Cx43 protein levels comparable with those of the control (Figure 4b). Furthermore, we used rapamycin, a potent inducer of autophagy that acts as a specific inhibitor of mTOR1, to corroborate our hypothesis. NMCs were treated for 48 h with Apelin-13 in the presence or absence of rapamycin. The results of the Western blot analysis showed that rapamycin was able to counteract the increase in Cx43 protein expression brought about by Apelin-13, thus preserving the Cx43 protein to the same levels as those in control cells (Figure 4d). Altogether, these results further support our hypothesis that, in NMCs, the increase in Cx43 mediated by Apelin-13 occurs through autophagy inhibition via the Akt/mTOR-dependent pathway.

## 3. Discussion

In this work, we proved for the first time that Apelin-13 increases Cx43 and gap junction function in NMCs by inhibiting autophagy through the activation of the Akt/mTOR signalling pathway.

It has been reported that altered connexin expression and gap junction remodelling are involved in many cardiac diseases such as atrial fibrillation and myocardial infarction, contributing to the predisposition to cardiac arrhythmias as well as to cell death [3,4]. Both in vitro and preclinical studies reported strong evidence that targeting Cx43, the prevalent gap junction isoform in the heart, may provide a path to ameliorate cardiac conductivity [4,8,29]. In addition, it has been shown through gene therapy approaches that the improvement in intracellular impulse propagation and the prevention of post-infarct arrhythmia occurrence could be reached by increasing Cx43 expression and/or by recovering the correct Cx43 distribution in the cardiac tissue [30,31,32]. Our present results demonstrate that Apelin-13 upregulates Cx43 protein expression and improves the ability of NMCs to form intercellular junctions and communicate with surrounding cardiomyocytes. In this scenario, we argue that the administration of Apelin-13 might exert a beneficial effect for the prevention of post-infarct arrhythmia similar to Cx43 targeting. Furthermore, this approach extends to the non-myocytic cardiac cell population as potential target cells that can benefit from this treatment.

We investigated this mechanism at a molecular level identifying that the process involved was the turnover of Cx43 protein rather than a transcriptional regulation of its gene. For this reason, we hypothesised a mechanism involving autophagy, which represents the main process of regulation and degradation for Cx43 [19]. The increased p62 level and reduction of the LC3II/LC3I ratio that we observed treating NMC cells with Apelin-13 were consistent with our hypothesis. Further confirmation came from the Cx43 up-modulation obtained by a 3-MA autophagy inhibitor. All these data suggest that Apelin-13 exerts its action on NMCs by inhibiting the autophagy flux. It is well-known that the AMP-activated protein kinase (AMPK) and Akt pathways are the main regulators of the autophagic process; however, while AMPK activation fosters this process [33], the Akt/mTOR signalling pathway decreases autophagy. Indeed, Akt after being phosphorylated by PI3K is able to target mTOR that, in turn, negatively regulates autophagy by inhibiting downstream autophagy-promoting proteins through phosphorylation [34]. In addition, the Akt/mTOR signalling pathway plays an essential role in protecting the heart [35]. We thus focused on this pathway, and the obtained results showed that Apelin-13 significantly increased both p-Akt/Akt and p-mTOR/mTOR already after 30 min of treatment. These data were also corroborated using Akt/mTOR pathway inhibitors, such as LY294002 and rapamycin, that effectively contrasted Apelin-13-induced Cx43 increase, thus confirming that, in NMCs, the increase in Cx43 brought about by Apelin-13 occurs through autophagy inhibition via the Akt/mTOR-dependent pathway. It had already been shown that Apelin-13 could interact with the Akt/mTOR signalling pathway in rat cardiomyocytes that undergone glucose deprivation [20,36,37], but for the first time, a similar mechanism is showed in NMCs. Conversely, in HL-1 cells, the involved pathway appeared to be different; indeed, it was reported that Apelin-13 triggered the inhibition of autophagy via AMPK-mTOR [26]. The different signalling cascades triggered by Apelin-13 might be attributed to the different cell types and/or the different pathological experimental models. This is in line with another study reporting that Apelin-13, used at high concentrations (2 µM) and for long time (4 days) in H9C2 cells, was able to induce hypertrophy through an autophagy increase via PI3K-Akt-ERK1/2-p70S6K [37]. In addition, other studies have reported that decreased levels of Cx43 were associated with an increase in the autophagic flux in neonatal cardiomyocytes and H9C2 cells [20,21,22].

The relevance of our results in the employed cellular model derives from the fact that NMCs are the largest population of cells in the heart and represent an important therapeutic target for diseased cardiac tissue. Indeed, despite the fact that cardiomyocytes form more than 70% of the heart volume of mammals, the NMCs occupying the remaining volume are more numerous than cardiomyocytes because of their small dimension [38]. Preservation of cardiac features and functionality is guaranteed by cardiomyocytes and non-myocyte populations that work closely together. It is known that by coupling adjacent cardiomyocytes, Cx43 plays a pivotal role in propagating the action potential; nevertheless, Cx43 is also expressed in NMCs, and growing evidence suggests that NMCs directly communicate with cardiomyocytes through Cx43 to exert specific functions [11]. For example, connexin-based signalling between endothelial cells and cardiomyocytes was found capable to synchronise cardiomyocytes and contribute to their spatial organisation and survival [39], whereas signalling between macrophages and cardiomyocytes facilitated atrioventricular node conduction [40]. In line with these physiological functions mediated by gap junctions, our results, evidencing an increase in the transport of Calcein-AM mediated by Cx43 up-modulation, were in line with this mechanism and support the hypothesis of a continuous communication between all cardiac cells. Functional coupling between cardiac fibroblasts and cardiomyocytes was also confirmed, both in vitro [41] and in situ [42], and a recent study provided the first direct proof of functional coupling between these two cell types in the whole heart [43]. The possible outcomes of such signalling are multifaceted. Several studies highlighted the possibility that cardiac fibroblasts may be involved in impulse transmission through gap junction-mediated electrical coupling with ventricular cardiomyocytes, specifically post myocardial infarction, to link infarct and non-infarct areas of the heart [44,45,46]. On the other hand, the possibility that connexin-based signalling between cardiac fibroblasts and cardiomyocytes might alter ventricular electrophysiology has been raised [47] and will deserve further investigation in biologically relevant samples.

Importantly, it has been reported that gap junction alteration in NMCs contributes to promoting arrhythmic events and adverse ventricular remodelling after myocardial infarction [11]. In addition, the downregulation of cardiac connexins can likewise trigger fibrosis [48,49,50,51]. Indeed, Cx43 downregulation and the consequent atrial gap junction interruption that characterise the atrial fibrillation condition are associated with an increase in fibrosis, which, in turn, further promotes conduction impairment and re-entry phenomenon development [25]. Therefore, therapeutic interventions targeting gap junctions in NMCs through exogenous Apelin delivery can be an appealing research path with considerable potential. A critical issue in this possible translational path might refer to the correct Apelin-13 dosage to be used, as some studies on this peptide pointed out that a high dose (>1 µM) of Apelin-13 can induce cardiac hypertrophy and stimulate autophagy [37], whereas lower doses induced cardioprotection [23]. The Apelin-13 dose (100 nM) that we optimised for NMC treatment was in line with the one used in pathological conditions for the recovery of the Cx43 protein level [25], thus suggesting that beneficial effects can be obtained with a low concentration of Apelin-13. Some studies have already shown that Apelin-13 ameliorated the Cx43 level in cardiomyocytes in in vitro experimental models that mimics pathological condition in which the expression of this protein was reduced, such as high glucose-induced downregulation in neonatal rat cardiomyocytes [25] and Angiotensin II-induced downregulation in HL-1 cells [26]. However, until now, the role of Apelin-13 on Cx43 remodelling in NMCs was unknown. Indeed, Cheng et al. reported that, in Apelin-13-treated HL-1 cardiomyocytic cells in the absence of Angiotensin II for 48 h, Cx43 expression remained unchanged in a wide range of concentrations tested [26]; in contrast, we observed a more than twofold enhancement of the Cx43 protein level in Apelin-treated NMCs at a 100 nM dose. These apparently contrasting results could reflect the cellular variability between the two studies, pointing out the potential important role played by NMCs in cardiac physiology. Nevertheless, our doses are in line with the results obtained in Apelin-13-treated neonatal cardiac myocytes [25]. Specifically, we speculated that treatment with Apelin-13 can represent a promising therapeutic strategy for those conditions where loss of Cx43 expression is associated with disease initiation and progression. This would be effective not only to avoid arrhythmias, via intercellular communication promotion between cardiomyocytes and NMCs, but also to reduce the formation of fibrous tissue. In addition, Apelin-13 treatment might be advantageous in improving the engraftment of implanted exogenous cells by fostering the formation of gap junctions with NMCs, thus overcoming the main limit of stem-cell therapy, such as a low persistence of injected cells in the infarcted hearts observed after a few days or weeks [52]. Further studies are needed to verify the effect of Apelin-13 in animal models exploring its potential therapeutic effect in pathological conditions such as myocardial infarction, with or without the injection of stem cells.

In conclusion, our study demonstrates the potential role of Apelin-13 in Cx-43 modulation and gap junction function in NMCs, the most abundant cardiac cell fraction. Apelin-13 promotes Cx43 expression in these cells via autophagy inhibition through the AKT/mTOR pathway. These results might pave the way for Apelin-13 as a candidate peptide for targeting gap junctions in NMCs.

## 4. Materials and Methods

### 4.1. Extraction of Cardiac Resident NMCs from Rat Hearts

Cardiac resident NMCs were isolated from the hearts of adult male Sprague–Dawley rats (4 months old, body weight 450–500 g). All animals received humane care in compliance with the Guide for the Care and Use of Laboratory Animals published by the US National Institutes of Health (NIH Publication n° 85–23, revised 1996) and in accordance with Italian law (DL-116, 27 January 1992). The scientific project was supervised and approved by the Italian Ministry of Health, Rome, and by the ethical committee of the University of Torino. Ten minutes after the intramuscular injection of heparin (1000 IU/kg body weight), rats were anaesthetised by the intraperitoneal injection of a mixture of ketamine (100 mg/kg) and xylazine (5 mg/kg). The absence of blink and paw withdrawal reflexes was checked before the rats were sacrificed. The rat heart was rapidly explanted, cannulated via the aorta, and retrogradely perfused using the Langendorff system, at a constant flow rate (5 mL/min) with a peristaltic pump for 5 min with oxygenated Ca^2+^-free Tyrode solution (154 mM NaCl, 4 mM KCl, 1 mM MgCl_2_, 5,5 mM D-glucose, and 5 mM HEPES, 1% Penicillin/Streptomycin, pH adjusted to 7.38) at 37 °C, in order to remove blood from the chambers. The hearts were subsequently perfused with 10 mL of oxygenated Ca^2+^ free-Tyrode containing Collagenase IV and Protease (1 mg/mL and 0.02 mg/mL, respectively) (Sigma-Aldrich, Saint Louis, MO, USA) and then with 30 mL of the same solution supplemented with 50 μmol/L CaCl_2_ to favour enzymatic tissue digestion. The hearts were detached from the cannula, atria were removed, and ventricles were minced in small fragments in 20 mL of Ca^2+^ free-Tyrode plus collagenase, protease, and 50 μmol/L CaCl_2_ solution. Fragments were gently mixed for three sequential passages in Ca^2+^ free-Tyrode plus Ca^2+^. The supernatant was collected and exposed to growing concentrations of CaCl_2_ (from 50 to 700 μM) to reach the same Ca^2+^ concentration of the culture medium. After centrifugation (1200 rpm, 10 min), NMCs were plated into 100 mm dishes and cultured in complete α-MEM (Sigma-Aldrich) containing 2 mmol/L L-glutamine, 100 U/mL penicillin, 100 μg/mL streptomycin, and 20% FBS (Sigma-Aldrich S.r.l.) at 37 °C and 5% CO_2_. After 24 h, cultured cells were washed twice with a physiological buffer solution in order to remove possible debris and cardiomyocytes, then new complete α-MEM containing 10% FBS was added and subsequently replaced every 3 days. NMCs were used for the experiments within passage 2.

### 4.2. Cell Culture and Drug Treatment

H9C2 cardiomyoblast cell line (purchased from the American Type Culture Collection, Manassas, VA, USA) was cultured in low glucose DMEM supplemented with 1% sodium pyruvate, 1% nonessential amino acids, 1% kanamycin, 1% L-glutamine, 0.1% β-mercaptoethanol, and 10% FBS (all from Sigma-Aldrich), and kept in an atmosphere of 5% CO_2_, 95% air at 37 °C in a humidified incubator. After reaching 80% confluence, cells were detached using Trypsin–0.2% EDTA (Sigma-Aldrich) and seeded at 4000 cells/cm^2^.

NMCs isolated from Sprague–Dawley rat hearts were used for the experiments at passage 1–2. NMCs were cultured in α-MEM (Sigma-Aldrich) containing 2 mmol/L L-glutamine, 100 U/mL penicillin, and 100 μg/mL streptomycin and supplemented with 10% FBS, and kept in an atmosphere of 5% CO_2_, 95% air at 37 °C in a humidified incubator. Cells were detached using Trypsin–0.2% EDTA (Sigma-Aldrich), and seeded at 5000 cells/cm^2^.

NMCs were treated with different concentrations of Apelin-13 (10 nM, 100 nM, or 1 µM, supplied by Cayman Chemical Company, Ann Arbor, MI, USA) for 30 min, 2, 4, 6, 16, 24, 48, or 72 h in α-MEM complete medium with 10% FBS.

NMCs were treated with the autophagy agonist rapamycin at 10 nM concentration (Sigma-Aldrich) in combination or not with 100 nM Apelin-13 for 48 h in α-MEM complete medium with 10% FBS or autophagy inhibitor 3-MA at the dose of 5 mM (Sigma-Aldrich) for 16 h (we chose this timing because a more prolonged treatment proved to be cytotoxic in these cells) in α-MEM 0% FBS after 2 h of culture in α-MEM 0% FBS. NMCs were pre-treated with a 25 μM phosphoinositide 3-kinase (PI3K) inhibitor LY294002 for 30 min and then treated with 10 μM LY294002 in combination or not with 100 nM Apelin-13 for 30 min, 2 or 48 h in α-MEM complete medium with 10% FBS.

### 4.3. Dye Transfer Assay

To monitor the diffusion of fluorescent molecules through gap junctions (dye coupling), we used a dye transfer assay. For this experiment, NMCs treated or not with 100 nM Apelin-13 for 48 h and loaded with the gap junction permeant dye, Calcein-AM (FITC) (250 nM) (Sigma-Aldrich), for 30 min were employed as donor cells, and H9C2 labelled with Did dye (APC) (1:5000) (supplied by Invitrogen, Thermo Fisher Scientific, Waltham, MA, USA) for 30 min (a dye that does not spread throughout cells) were used as acceptor cells. Did dye was used for discerning the two cell types after the dye transfer. In some experiments, in order to demonstrate that Calcein-AM transfer occurs only through the gap junction, NMCs were preincubated with a specific gap junction communication blocker, i.e., carbenoxolone-disodium (CBX) (100 μM) (Sigma-Aldrich), overnight before carrying out the previously reported experimental protocol. All treatments were performed in an atmosphere of 5% CO_2_, 95% air at 37 °C in a humidified incubator.

After labelling, cells were co-cultured (NMCs/H9C2 ratio 2:1) for 24 h at 37 °C in a humidified incubator, and APC+FITC+ double-positive cells were analysed through flow cytometry. Cells were acquired with a CyAN ADP flow cytometer (Beckman Coulter, Brea, CA, USA) and analysed by FlowJo^®^ software (BD Biosciences, V10). At least 80,000 events per sample were collected.

### 4.4. Immunofluorescence and Confocal Microscopy

Adhered NMCs at the end of the experiments were processed for confocal immunofluorescence. Cells were seeded on coverslips and fixed with 4% PFA, permeabilised with 0.1% Triton X-100, when necessary, and blocked with 5% (*w/v*) BSA and 2.5% (*v/v*) normal goat serum (NGS) for 30 min. NMCs were then stained overnight at 4 °C with the anti-Apelin receptor (APJ) rabbit primary antibody (1:50, by Santa Cruz Biotechnology, Santa Cruz, CA, USA), anti-connexin-43 rabbit primary antibody (1:400, purchased from Sigma-Aldrich), anti-LC3B rabbit primary antibody (1:100, from Cell Signaling Technology, Danvers, MA, USA), and anti-TnC mouse primary antibody (1:50, purchased from Santa Cruz Biotechnology) in 1× PBS. Cells were then incubated for 30 min at room temperature (RT) with Alexa Fluor 488 goat anti-rabbit secondary antibody (1:1000, supplied by Invitrogen, Thermo Fisher Scientific) to visualise the APJ Receptor and LC3B, with Alexa Fluor 647 goat anti-rabbit secondary antibody (1:1000, supplied by Invitrogen, Thermo Fisher Scientific) to observe connexin-43, and with Alexa Fluor 546 goat anti-mouse secondary antibody (1:1000, purchased by Invitrogen, Thermo Fisher Scientific) to see TnC. Nuclear staining was performed with Hoechst-33342 (1.5000, Sigma-Aldrich). Stained NMCs were analysed with an inverted confocal laser-scanning microscope LSM 800 (Carl Zeiss Inc., Oberkochen, Germany). Quantification of Cx43 was performed using ImageJ^®^ software (USA, http://rsb.info.nih.gov/ij/), as we previously described [53].

### 4.5. Western Blot Analysis

NMCs were lysed with a RIPA buffer supplied with protease and phosphatase inhibitors (from Sigma-Aldrich). Extracted proteins were quantified through the BCA assay (Thermo Fisher Scientific), and 15 μg total proteins were loaded for each sample. SDS-PAGE (12% Bis-Tris gel, from Invitrogen, Thermo Fisher Scientific) and blotting on polyvinylidene difluoride transfer membranes (GE Healthcare Life Science, Chicago, IL, USA) were performed. Membranes were blocked in 5% milk or 5% BSA in 1× PBS for 1 h at RT and probed with the primary antibodies (anti-β-Actin, anti-connexin-43, anti-p62, and anti-Vinculin purchased from Sigma-Aldrich; anti-LC3B, anti-P-Akt, anti-Akt, anti-P-Ampk, and anti-Ampk from Cell Signaling Technology; anti-P-mTOR supplied by Abcam; anti-mTOR by Bethyl Laboratories, Inc., Montgomery, TX, USA; anti-APJ and anti-TnC by Santa Cruz Biotechnology) 1:1000 overnight at 4 °C, followed by secondary antibodies (1:5000, from Invitrogen, Thermo Fisher Scientific) for 1 h at RT. Bands were visualised using a chemiluminescence kit (Clarity western ECL substrate, from Bio-Rad Laboratories, Hercules, CA, USA) and ChemidocTouch (Bio-Rad). Quantification was performed using Image Lab Software (Bio-Rad Laboratories).

### 4.6. RNA Extraction, cDNA Synthesis and Quantitative Reverse Transcriptase PCR (RT-qPCR)

NMCs were treated with Trizol reagent (Life Technologies, Carlsbad, CA, USA), and total RNA (totRNA) was extracted according to the manufacturer’s instructions. Genomic DNA contaminations were removed by DnaseI treatment (purchased by Ambion, Thermo Fisher Scientific). Subsequently, 1 µg of totRNA was retrotranscribed with random hexamer primers using a High Capacity Reverse Transcription Kit (Applied Biosystems, Waltham, MA, USA) in accordance with the manufacturer’s suggestions. The expression levels of target genes were evaluated with SYBR green technology on an ABI PRISM 7500 Fast Real-Time PCR system (Applied Biosystems) using 25 ng of equivalent RNA as template and 150 nM of each primer.

Employed primers were Gap Junction Alpha-1 (*Gja1*) (connexin-43) (RefSeq: AY324140.1): forward primer 5′-AGTACGGGATTGAAGAGCAC-3′; reverse primer 5′-GTGAGAGGAAGCAGTCTACC-3′ and glyceraldehyde-3-phosphate dehydrogenase (GAPDH) (RefSeq: AF106860.2): forward primer 5′-CAAGTTCAACGGCACAGTCAAG-3′; reverse primer 5′-GGTGGTGAAGACGCCAGTAGA-3′.

Melting curve analysis was performed for all amplicons. Expression levels of *Gja1* in NMCs treated with Apelin-13 and untreated cells were indicated as log2(*Gja1*/GAPDH).

### 4.7. Statistical Analysis

Data were expressed as means ± standard errors of the mean (SEM) of at least three independent experiments. Statistical comparisons were performed with paired or unpaired Student’s *t*-test. Differences with *p* ≤ 0.05 were regarded as statistically significant.

## Data Availability

Data are contained within the article.
